# Dental Management of Glanzmann's Thrombasthenia in a 4‐Year‐Old Child With Rampant Caries: A Case Report and Literature Review

**DOI:** 10.1002/ccr3.71513

**Published:** 2025-11-25

**Authors:** Bahareh Nazemisalman, Gisoo Bahmani, Kambiz Davari, Mobina Sadat Zarabadi

**Affiliations:** ^1^ Department of Pediatric Dentistry, School of Dentistry Zanjan University of Medical Sciences Zanjan Iran; ^2^ Student Research Committee, Department of Pediatric Dentistry, School of Dentistry Zanjan University of Medical Sciences Zanjan Iran; ^3^ Department of Pediatrics, School of Medical Sciences Zanjan University of Medical Sciences Zanjan Iran; ^4^ USERN Office, Qazvin University of Medical Sciences Qazvin Iran

**Keywords:** bleeding disorder, caries, children, dentistry, Glanzmann's Thrombasthenia

## Abstract

Glanzmann's Thrombasthenia (GT) is a rare congenital bleeding disorder in children, caused by the absence or dysfunction of glycoprotein (GP) IIb/IIIa, a receptor located on the platelet membrane. Proper oral health and regular dental appointments are crucial for these patients. However, maintaining good oral hygiene is challenging among them. Due to excessive gingival bleeding, patients refrain from brushing their teeth, which leads to poor oral hygiene and severe dental caries. Furthermore, gingival inflammation caused by dental plaque may exacerbate the bleeding and create a vicious cycle. A 4‐year‐old girl with the chief complaint of pain and discomfort in the upper left deciduous molars was examined in April 2024. According to her past medical history, she was previously diagnosed with GT. She was the firstborn child of parents of a consanguineous marriage with no other siblings. Physical examination revealed petechiae and ecchymosis on her face, trunk, abdomen, legs, and limbs that resolved spontaneously over time. Clinical intraoral examination revealed rampant dental caries and generalized gingivitis. The patient was hospitalized 1 day before the surgery and received 1 dose of Recombinant Activated Factor VII preoperatively. Dental caries were removed and restored under general anesthesia. However, 1 week after the dental operation, the patient experienced sudden and severe bleeding, requiring two units of blood transfusions at that time. Dental care for patients with GT might be challenging due to the risk of excessive bleeding. It's essential to consider platelet transfusions in case of a significant risk of bleeding during surgery, and with extreme caution. Furthermore, severe delayed bleeding requiring transfusion as a postoperative complication should be considered and managed. Dental procedures should be performed with minimal tissue trauma to prevent bleeding.

## Key Clinical Message

- Dental management of patients with GT might be challenging due to the risk of excessive bleeding.
- Dental procedures should be performed with minimal tissue trauma to prevent bleeding.
- It is essential to educate patients and caregivers on proper oral hygiene practices to prevent future dental complications.

## 1 Introduction

Glanzmann's Thrombasthenia (GT) is a rare congenital bleeding disorder in children, first described by Eduard Glanzmann in 1918 [1]. GT is caused by the absence or dysfunction of glycoprotein (GP) IIb/IIIa which is located on the membrane of platelets [2]. The disorder is characterized by a normal platelet count and morphology, but impaired platelet aggregation and clot retraction which lead to prolonged bleeding time [3]. Patients are often detected early at a young age, presenting with symptoms of easy bruising and mucocutaneous bleeding [4]. The severity and frequency of bleeding episodes can be quantified using Bleeding Assessment Tools (BAT) and the bleeding score [5]. The typical clinical manifestations include mucocutaneous bleeding such as purpura, epistaxis, and gingival hemorrhage. In addition, patients may experience gastrointestinal hemorrhage and heavy menstrual bleeding [6]. GT affects around 1 in every 1,000,000 individuals [4]. Certain ethnic groups such as Iraqi Jews, Palestinian Arabs, and French Gypsies have a higher prevalence of Glanzmann due to a high rate of consanguineous marriages [7].

GT is inherited in an autosomal recessive pattern, with the mutations affecting ITGA2B or ITGB3 genes which are located closely on chromosome 17q21.31–32. These genes encode αIIb and β3 [8]. ITGA2B has a higher frequency of pathogenic variants compared to ITGB3, presumably because it has 30 exons compared to ITGB3 which has 15 exons [2]. IIb/IIIa is a heterodimeric molecular complex consisting of two subunits (αIIb and β3) with a non‐covalent link, enabling duplex signaling between the cell membrane and the extracellular matrix region as well as intracellular signaling [3]. The IIb‐IIIa also plays an important role as a receptor that induces platelet aggregation [9].

As mentioned before, clinical features include purpura as one of the primary manifestations in newborns, epistaxis and gingival bleeding as the most common signs among children, mucocutaneous bleeding, easy bruising, severe hemorrhage following minor trauma, and menorrhagia. Other manifestations such as hematuria, gastrointestinal hemorrhages, and intracranial hemorrhage may occur in some cases [1, 3]. However, bleeding severity varies among the patients [1]. Although Glanzmann is a serious bleeding disorder, proper medical care ensures a good prognosis for the disease [10].

The management of GT requires a multidisciplinary approach among the medical team including a surgeon, anesthetist, hematologist, pharmacist [11]. For minor bleedings, the initial management comprised local pressure, cauterization, suturing, and the administration of tranexamic acid and other antifibrinolytic agents. In patients with severe bleeding, platelet transfusion or recombinant activated clotting factor VII might be necessary [12].

The oral cavity has a rich blood supply because of its high vascularity; due to the limited space in the operating field, achieving proper surgical hemostasis in this area can be challenging [13]. Excessive bleeding following dental extraction might be the early sign of the disease [14]. Gingival bleeding may occur due to gingivitis and poor oral hygiene; therefore daily flossing is recommended [4]. Primary teeth exfoliation might be a common cause of bleeding during childhood [4]. Proper oral health and regular dental appointments are crucial for these children, and dental procedures should be performed with minimal tissue trauma and without any bleeding if possible [15]. Moreover, antifibrinolytics in the form of mouthwash, plastic splints, and fibrin glue might help prevent severe bleeding [4].

Due to the rarity of GT, randomized clinical trials are limited, making it difficult to establish management guidelines [16]. According to platelet dysfunctions, excessive and severe bleeding may occur at any time, so immediate management by an expert clinician is required [11].

## 2 Case Presentation

### 2.1 Patient Information and Timeline

A 4‐year‐old girl with the chief complaint of pain and discomfort in the upper left deciduous molars was examined in April 2024. According to her past medical history, she was a previously diagnosed case of GT. She was the firstborn child to parents in a consanguineous marriage with no other siblings. There was no history of Glanzmann or other similar conditions in her family. The disorder was first diagnosed at 6 months of age following severe epistaxis based on multiple blood tests and clinical findings. Tranexamic acid capsules were administered four times daily as part of the patient's routine medication regimen. In addition, the patient took daily supplements consisting of folic acid, vitamin B12, zinc, and iron. Her medical history indicated multiple hospitalizations for recurrent episodes of epistaxis, and she received recombinant activated factor VII (AryoSeven) as a treatment. Previous laboratory tests demonstrated prolonged bleeding time, normal platelet counts, and lack of platelet aggregation in response to adenosine diphosphate, collagen, and arachidonic acid, while aggregation of Ristocetin was normal. The hematology tests indicated low levels of M.H.C, M.C.H.C, and hypochromic red blood cells (Tables S1–S4).

### 2.2 Clinical Findings and Diagnostic Assessment

**FIGURE 1:**
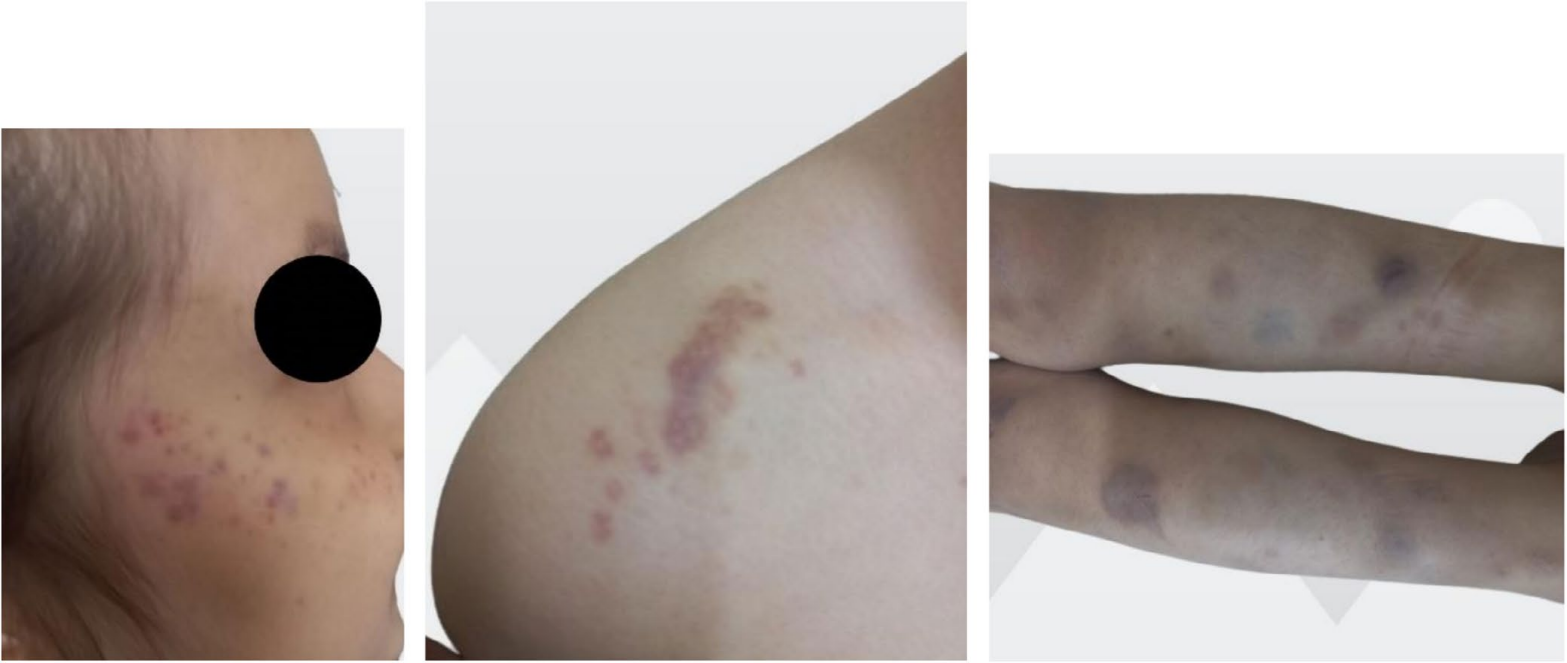
Petechiae and ecchymosis on different parts of the body.

**FIGURE 2:**
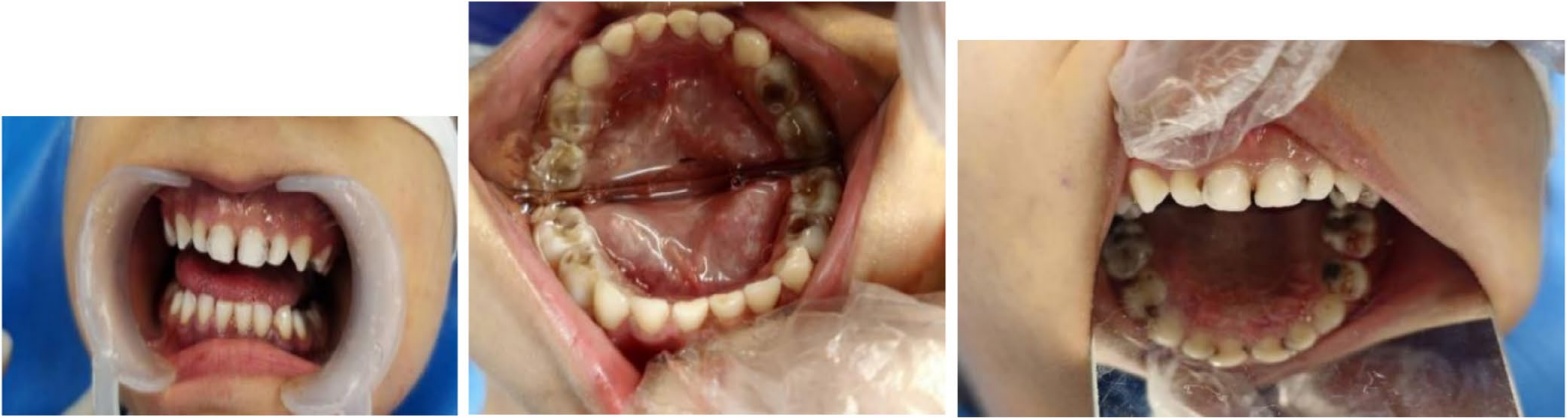
Preoperative oral examinations. Multiple rampant caries present in anterior and posterior teeth.

Physical examination revealed petechiae and ecchymosis on her face, trunk, abdomen, legs, and limbs, which resolved spontaneously over time (Figure 1). Clinical intraoral examination revealed poor oral hygiene and generalized gingivitis. According to her parents, they avoided brushing her teeth due to fear of excessive bleeding, which resulted in poor oral hygiene and several dental caries. Multiple carious lesions were observed in the anterior and posterior teeth (Figure 2). The patient wasn't cooperative in taking dental X‐rays. Given the patient's condition and lack of cooperation, dental treatment under general anesthesia was deemed the most appropriate option.

### 2.3 Therapeutic Intervention

**FIGURE 3:**
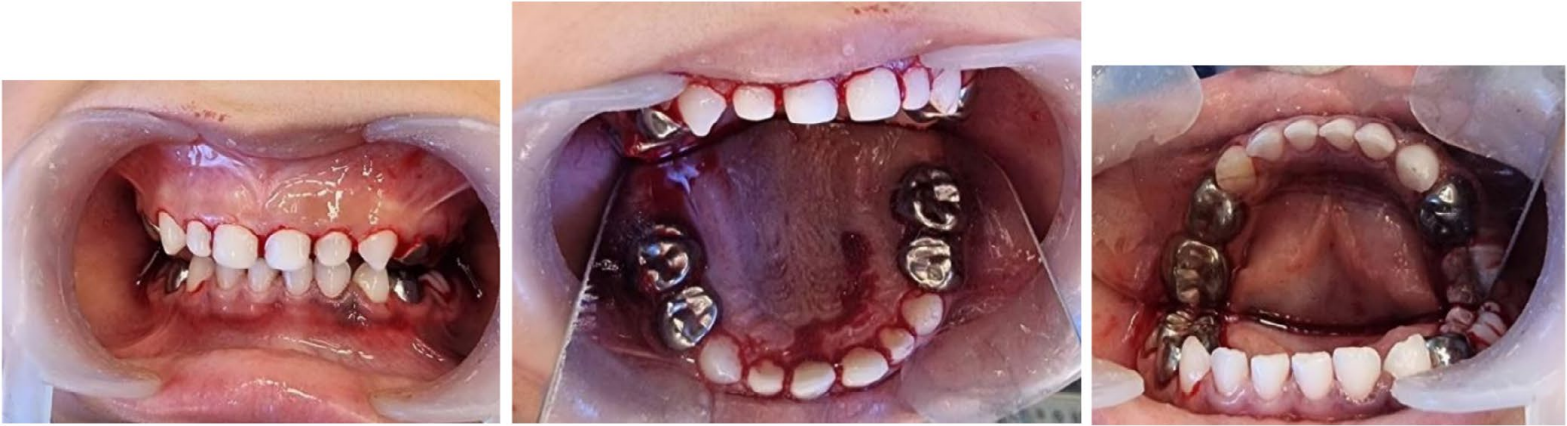
Maxillary and mandibular teeth post‐operation.

Prior to any interventions, laboratory tests were requested. Prothrombin time was within the normal level (12.3 s), and International Normalized Ratio (INR) was 0.9. The patient was hospitalized 1 day before surgery and received a single preoperative dose of Recombinant Activated Factor VII (AryoSeven 2.4 mg (120 KIU) powder and solvent for solution for injection, Iran). For the nasal intubation, the smallest available endotracheal tube diameter was selected to prevent epistaxis. Pulpectomy was performed using calcium hydroxide temporary filling material (Metapex Plus, Meta Biomed, South Korea) followed by restoration with composite resin (Estelite Posterior Tokoyama PA1, Japan) as the elective treatment for the maxillary anterior teeth (Figure 3). Molars were treated with pulpotomy using modified accelerated setting zinc oxide eugenol cement (Zonalin Kemdent, England) and bioceramic reparative cement (MTA Angelus, Brazil). Stainless steel crowns (MIB, South Korea) were placed on the mentioned teeth (Figure 3), except for the primary mandibular second molar, which was restored with composite resin (Estelite Posterior Tokoyama PA1, Japan). Topical fluoride varnish was applied as a preventative approach (Table 1).

**TABLE 1:** Therapeutic interventions under general anesthesia.

Number of teeth	Therapeutic interventions
75^a^	Amalgam restoration
54, 55, 64, 65, 74, 84, 85	Pulpotomy + SSC
51, 52, 53, 61, 62	Pulpectomy + composite resin restoration
All teeth	Topical fluoride varnish

^a^ Two‐digit numbering system. The first digit indicates the quadrant of the tooth, while the second digit indicates the position of the tooth from the midline of the arch.

**FIGURE 4:**
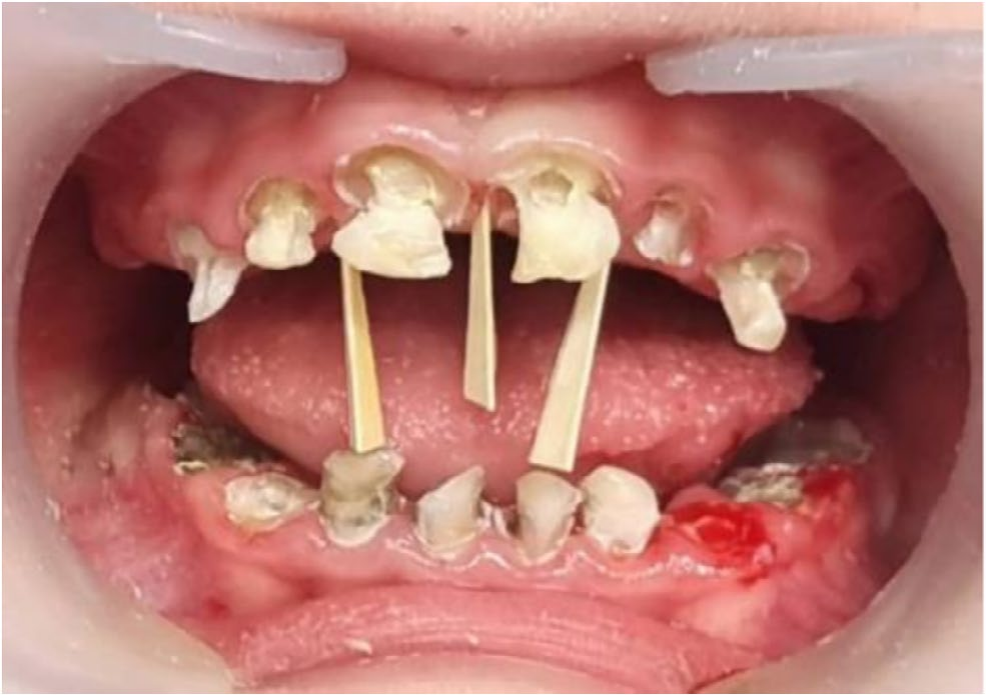
Wooden wedges were physically pressed against the bleeding point to compress the vessels and control the bleeding.

During the surgery, dental wooden wedges were used to control bleeding (Figure 4). Parents were advised to provide the child with a soft diet for a week and to manage the child's dental plaque using a toothbrush or by scrubbing with gauze.

### 2.4 Follow‐Up

One week after the dental procedure, the patient experienced sudden and severe bleeding, especially in the left maxillary region, leading to a drop in hemoglobin levels below 8 g/dL. As a result, she received two units of blood transfusions 1 week after the surgery. All mentioned steps are available in Table 2.

**TABLE 2:** All steps from primary examination to follow‐up in a glance.

**In Dental office**	
1. Chief complaint	4‐year‐old girl with pain and discomfort in upper left deciduous molars
2. Medical history	GT diagnosed at 6 months; recurrent epistaxis, treated with Tranexamic acid and recombinant activated factor VII
3. Physical examination	Petechiae, ecchymosis on face, trunk, abdomen, limbs; poor
4. Oral examination	Oral hygiene, gingivitis, multiple carious lesions in anterior and posterior teeth in both jaws
5. X‐ray evaluation	No dental X‐rays were taken (uncooperative patient)
6. Treatment planning	Treatment under general anesthesia due to patient's condition and noncooperation
**Pre‐operation**	
7. Preoperative laboratory tests	PT (12.3 s), INR (0.9)
8. Preoperative medication	Administer 1 dose of Recombinant Activated Factor VII (AryoSeven 2.4 mg)
**During operation**	
9. Nasal intubation	Use smallest diameter endotracheal tube to prevent epistaxis
10. Elective treatment for anterior teeth	Pulpectomy with calcium hydroxide followed by composite resin restoration
11. Elective treatment for molars	Pulpotomy with zinc oxide eugenol cement and bioceramic cement (MTA Angelus), followed by stainless steel crowns
12. Preventative approach	Apply topical fluoride varnish
13. Intraoperative hemostasis	Use dental wooden wedges to control bleeding. Wooden wedges were physically pressed against the bleeding point to compress the vessels and control the bleeding
**Post‐operation**	
14. Postoperative care	Soft diet for 1 week; dental plaque control with toothbrush or gauze
15. Follow‐up (1 week)	Severe bleeding, hemoglobin drop below 8 g/dL, received 2 units of blood transfusion

Despite consistent efforts to ensure follow‐up dental care, the patient was not cooperative with scheduled appointments, due to residing in a different city. According to reports from her hematologists, she was hospitalized 11 times between April 2024 and May 2025 for recurrent episodes of epistaxis. Each hospitalization necessitated the administration of RBC transfusions as part of her treatment. Her medication regimen has remained stable to date.

## 3 Discussion

GT is an inherited disorder characterized by defects in the αIIbβ3 GP, which is located in the platelet membrane [11]. This protein is essential for platelet aggregation; thus, the absence or dysfunction of the αIIbβ3 integrin compromises vascular integrity in the case of trauma or results in episodes of spontaneous mucocutaneous bleeding in patients [8, 11]. There are three types of GT, as described: (1) type I with less than 5% residual IIb/IIIa, (2) type II with 5%–20% residual IIb/IIIa, and (3) type III with more than 20% IIb/IIIa which is rare [8]. Type I and II represent quantitative abnormalities, while type III (also called variant GT) is defined by qualitative abnormalities [17]. Nonfunctional αIIbβ3 leads to the inability of platelets to bind fibrinogen or other adhesive proteins, leading to a loss of thrombus formation following vessel injury and deficient clot retraction in many cases [9]. Acquired GT has also been reported in some cases and is associated with autoantibodies or paraproteins that inhibit platelet aggregation without reducing the platelet count. This condition results from the spontaneous inhibition of normally expressed αIIbβ3 [18].

Most patients are diagnosed before the age of five, and are characterized by recurrent episodes of epistaxis and gingival bleeding, which are among the most prevalent clinical manifestations [3]. The diagnosis of GT requires a normal platelet count, prolonged bleeding time, and an elevated PFA (Platelet Function Analyzer). Furthermore, a unique diagnostic feature is that platelets fail to aggregate in Light Transmission Aggregometry (LTA) [3]. For an accurate diagnosis, it is important to choose appropriate laboratory tests [3]. The unique laboratory feature of GT is characterized by the absence of platelet aggregation in response to all physiological agonists, except a normal response to Ristocetin [19]. In this case, platelet aggregation tests confirmed the diagnosis at the age of 6 months. The tests demonstrated a lack of platelet aggregation in response to adenosine diphosphate, collagen, and arachidonic acid, but a normal response to Ristocetin. Additionally, there was a history of consanguineous marriage in the parents. The main reason for repeated hospitalizations was the recurrence of epistaxis. Bleeding might be unpredictable in GT, therefore, the initial management involves platelet transfusions and antifibrinolytic agents, particularly during bleeding episodes or prior to invasive procedures [20]. However, in this case, despite receiving Recombinant Activated Factor VII preoperatively, severe bleeding presisted for 1 week after surgery, resulting in hemoglobin levels dropping below 8 g/dL.

Management of GT initially involves preventive care [21]. Medications that affect platelet functions such as aspirin and NSAIDs must be avoided, and due to the risk of infection associated with frequent transfusions, hepatitis B vaccination should be considered [21]. In addition, regular dental visits are recommended to prevent gingivitis and oral bleeding. Iron supplements can help prevent iron deficiency anemia, which is often associated with chronic bleeding in these patients [21]. A hematologic evaluation is essential before any invasive procedure in GT patients, and platelet transfusions should be readily available in cases where surgical bleeding is anticipated [13]. Other interventions, such as compression, gelatin sponges, antifibrinolytic agents, and YAG lasers, can be employed to control minor bleeding effectively [14]. The patient's hemoglobin level should be monitored daily after surgery. A soft diet and meticulous oral hygiene are also recommended during the recovery period [13]. Additional bleeding management strategies, such as desmopressin, immunoabsorption, and allogeneic bone marrow transplantation, have also been used, although they either lack sufficient clinical efficacy or are not universally available [14]. In the present case, intraoperative bleeding was managed by optimizing coagulation factors preoperatively and administering factor VII. Recombinant activated factor VII promotes hemostasis in severe bleeding conditions, such as GT, serving as a preventive treatment to avert bleeding incidents. It activates thrombin, which then converts fibrinogen to fibrin and activates platelets, forming a haemostatic plug [22]. At pharmacological doses, recombinant activated factor VII (rFVIIa) induces a thrombin burst by directly binding to activated platelets; therefore, it is essential to have an adequate platelet count. In patients with GT, although the platelet count is sufficient, there is impaired platelet aggregation, which is why rFVIIa is utilized. Several case studies have documented successful control of bleeding following the administration of rFVIIa at doses ranging from 80 to 120 μg/kg in surgical settings [23]. However, due to the short half‐life of rFVIIa (2–4 h) [24] and the tissue sensitivity, along with the inflammation that often accompanies surgical procedures, postoperative bleeding can occur., necessitating the transfusion of two units of blood for our patient.

Gingival bleeding is primarily caused by poor oral hygiene and local factors [25]. In our case, the child's parents avoided brushing her teeth due to fear of oral bleeding, which resulted in poor oral hygiene. Research indicates that children with bleeding disorders experience considerable dental issues such as poor oral hygiene and high numbers of carious teeth, which may lead to a risk of health complications [26, 27, 28]. To maintain gingival health, it is essential to educate patients and caregivers on proper oral hygiene practices to prevent future dental complications [29]. The recommended duration for tooth brushing should be at least 2 min, twice a day, and it is encouraged to regularly replace toothbrushes and utilize disclosing tablets to identify areas that have not been brushed properly [30]. Furthermore, the use of dental floss and interdental brushes is beneficial in preventing dental caries and periodontal disease [31]. Toothbrushes with medium bristles are recommended for these patients; hard bristles may cause abrasions, while soft bristles may be ineffective in removing plaque [31]. Fluoride toothpaste with a concentration of 1000 ppm is suitable for children under 7 years old, while 1400 ppm is appropriate for those over 7. Fluoride supplements are not recommended when the water supply contains fluoride levels of 1–1.5 ppm or higher, due to the risk of dental fluorosis or thyroid hormone insufficiency [31]. Regular dental follow‐ups and fluoride varnish applications four times a year are recommended [30]. In our case, fluoride varnish was applied at the final treatment stage to prevent future caries. Research indicates that professionally applied fluorides are effective in preventing and controlling dental caries, particularly in low‐ and middle‐income countries [32]. Future studies should explore the efficacy of newly developed remineralizing agents, such as biomimetic hydroxyapatite toothpaste and casein phosphopeptide‐amorphous calcium phosphate [33, 34]. Particularly in special‐care populations.

When treating patients with congenital bleeding disorders, care should be taken to avoid trauma to the oral mucosa. Sharp edges in restorations or removable prosthetics should be eliminated [35]. Rubber dams are recommended to protect soft tissues during management [29]. Dental appointments should be scheduled as close as possible to the time of hemostatic agents administration [35]. The use of suction tips should be avoided to prevent any bruises to mucosal injury; cotton rolls may be used as an alternative [14]. Previous case reports are summarized in Table 3.

**TABLE 3:** Previous case reports and case series of dental management in patients with GT.

Author	Year	Type of article	Number of participants	Age	consanguineous marriage	Medical condition	Dental condition	Procedure	Preoperative intervention	Postoperative intervention
Jairam et al. [36]	2022	Case report	1	4	+	GT	Discolored anterior tooth due to trauma	Pulpectomy	A cotton pellet soaked with BotroClot solution into the canal	NR
Bhavyaa et al. [37]	2021	Case report	1	7	+	GT	Spontaneous oral bleeding	Extraction Restoration with SSC^a^	Two units of platelet and two units of packed cell volume transfusions Tranexamic acid (mouthwash)	1/2 unit of platelets Tranexamic acid (powdered) in socket Paracetamol (IV) Tranexamic acid (IV, in soft splint)
Rakesh et al. [38]	2019	Case report	1	3	−	GT	Spontaneous gingival bleeding due to dental trauma	Rigid splint	Hemocoagulant at home	Topical hemocoagulant
Félix et al. [39]	2019	Case report	1	NR	NR	GT	NR	Periodontal surgery Extraction	1 Pack of platelet concentrate Tranexamic acid (oral)	Tranexamic acid (oral)
Venkat et al. [25]	2018	Case report	1	10	−	GT	Spontaneous gingival bleeding and caries	Oral prophylaxis	Three units of blood transfusion Livogen and tranexamic acid	Vitamin C supplements Soft tooth brush with fluoridated toothpaste
Prud'homme et al. [40]	2018	Case report	1	5	NR	GT	Localized aggressive periodontitis	Extraction Scaling and root planning	Recombinant Factor VIIa injection	Tranexamic acid
Segna et al. [13]	2016	Case series	3	8	NR	GT	Recurrent episodes of gingival bleeding due to radicular cyst	Extraction	Tranexamic acid IV	Hemostatic gauze in socket Tranexamic acid (mouthwash) Chlorhexidine (mouthwash) Antibiotic prophylaxis
				13	NR		Odontogenic pain	Extraction	Tranexamic acid IV	Hemostatic cellulose gauze in the socket Tranexamic acid (mouthwash) Chlorhexidine (mouthwash) Antibiotic prophylaxis
				24	NR		Severe gingival bleeding	Extraction	1 unit of platelets and tranexamic acid 1 unit of red blood cells	Second blood transfusion Tranexamic acid (oral, IV and mouthwash) Chlorhexidine (mouthwash) Antibiotic prophylaxis
Ghosh et al. [41]	2016	Case report	1	18	+	GT	Generalized gingivitis and chronic spontaneous bleeding	Total extraction	NR	Tranexamic Acid (powder in the socket) 4 Units packed cells (red blood cells) 34 Units platelet rich concentrates Injection of Recombinant coagulation factor VIIa (in 2 weeks)
Gopalakrishnan et al. [42]	2014	Case report	1	11	−	GT	Hard swelling on the maxillary arch	Cyst enucleation and extraction	Tranexamic acid 500 mg Transfusion of seven units of platelets and 1 unit of packed red blood cells	Transfusion of 3 units of platelets Tramadol (pain control) Tranexamic acid (mouthwash)
Varkey et al. [15]	2013	Case report	1	4	−	GT	Gingival bleeding and swelling	Restoration	1 unit of platelet transfusion	Antibiotic prophylaxis
N Mehta et al. [14]	2010	Case report	1	6	+	GT Seizure disorder	Caries and peri‐radicular bone loss	Restoration Extraction	NR	Acrylic‐splint and Calgigraf Ag‐Foam 10 days postoperatively
Ranjith et al. [43]	2008	Case report	1	22	+	GT	Severe spontaneous gingival bleeding	Supra and subgingival Scaling and root planning	Platelet transfusion	Anti‐plaque agents
Toygar et al. [44]	2007	Case series	2	11	+	GT	Spontaneous gingival bleeding	Scaling and root planning Removing blood clots	1 Unit of platelet transfusion	Chlorhexidine 0.2% (mouthwash)
				26	+		Excessive gingival bleeding	Removing plaque, fresh blood, and blood clots	Three units of platelet transfusion	Chlorhexidine 0.2% (mouthwash)
Gomes et al. [45]	2004	Case report	1	6	NR	GT	Caries and periapical lesion Moderate gingivitis	Extraction	Platelet‐concentrate transfusion Epsilon aminocaproic acid	Epsilon aminocaproic acid

Abbreviation: NR, not reported.

When conservative measures are ineffective, platelet transfusions remain the standard therapeutic and prophylactic approach for GT. However, this strategy carries risks, including pathogen transmission, alloimmunization, and platelet refractoriness [46]. The administration of Recombinant Activated Factor VII represents a promising alternative for managing severe bleeding episodes [46]. Recombinant Factor VII shortens clotting time, reduces the time to peak thrombin generation, and enhances the maximum rate of thrombin generation [47]. Inferior alveolar nerve blocks pose a hematoma formation risk and should be replaced with infiltration or intra‐ligament anesthesia in these patients [29]. Factor replacement therapy and general anesthesia may be necessary for invasive dental procedures [29]. As described in Table 3, most studies employed platelet transfusions and tranexamic acid administration as preoperative interventions to prevent bleeding. Additionally, tranexamic acid and chlorhexidine mouthwash were used as postoperative interventions in the majority of studies. However, some studies also included blood transfusions, packed red blood cells, and factor VII administration in the postoperative phase [13, 41, 42]. Notably, one study incorporated the use of an acrylic splint and Calgigraf Ag‐Foam 10 days after the procedure [14]. Aminocapric acid was utilized as a hemostatic agent in the postoperative care of patients in one study as well [45].

Our biggest limitation was the inability to accurately assess all cavities and dental conditions before administering general anesthesia due to excessive gingival bleeding triggered by the minimal contact with a dental explorer or dental mirror, combined with the child's uncooperative behavior, which ultimately prevented us from obtaining the dental radiographs. During general anesthesia, significant bleeding occurred despite the preoperative administration of recombinant activated factor VII, complicating the surgical procedure. This made it challenging to differentiate between pulpal bleeding and pathological bleeding. To manage the hemorrhage, we employed gingival retraction cords, wooden wedges, gelfoams, epinephrine, and other hemostatic measures. Even after the dental procedures were completed, the patient experienced episodes of sudden, severe bleeding requiring hospitalization and blood transfusions.

## 4 Conclusion

Dental care in patients with GT can be challenging due to the risk of excessive bleeding. A multidisciplinary team, including a pediatrician, an anesthesiologist, and a hematologist, should collaborate to ensure the child's well‐being. It's important to have platelet transfusions available in case of a significant risk of bleeding during surgery, and to perform surgery with great caution to ensure effective hemorrhage control to minimize complications.

## Author Contributions

**Bahareh Nazemisalman:** conceptualization, data curation, funding acquisition, investigation, methodology, project administration, resources, supervision, validation, writing – original draft, writing – review and editing. **Gisoo Bahmani:** conceptualization, data curation, methodology, visualization, writing – original draft, writing – review and editing. **Kambiz Davari:** conceptualization, data curation, funding acquisition, investigation, methodology, project administration, supervision, validation. **Mobina Sadat Zarabadi:** conceptualization, methodology, project administration, supervision, validation, visualization, writing – original draft, writing – review and editing.

## Funding

The authors have nothing to report.

## Ethics Statement

A written consent was obtained from parents to publish the medical and dental information without declaring the patient's identity.

## Conflicts of Interest

The authors declare no conflicts of interest.

## Supporting information


**Table S1:** Platelet aggregation and coagulation tests results.
**Table S2:** Hematology tests results.
**Table S3:** Hematological tests before surgery.
**Table S4:** Coagulation tests.

## Data Availability

The findings of this case report are backed up by the data (figures and tables), which are included in the manuscript and can be provided upon request.
