# Supplementary material for: Dental Management of Glanzmann's Thrombasthenia in a 4‐Year‐Old Child With Rampant Caries: A Case Report and Literature Review
Source: Clin Case Rep. 2025 Nov 25;13(12):e71513. doi: 10.1002/ccr3.71513 (PMC12646865; doi:10.1002/ccr3.71513)
Supplement: Supplementary file 1 — Table S1: Platelet aggregation and coagulation tests results. Table S2: Hematology tests results. Table S3: Hematological tests before surgery. Table S4: Coagulation tests. [file CCR3-13-e71513-s001.docx]

| Test | Result | Unit | Normal range |
| --- | --- | --- | --- |
| Ristocetin 0.75 mg/ml | 12 | % | 8-35 |
| Ristocetin 100 mg/ml | 24 | % | 16-70 |
| Ristocetin 1.25 mg/ml | 49 | % | 32-100 |
| Ristocetin 1.50 mg/ml | 68 | % | 50-150 |
| **Platelet aggregation (ADP)** |  |  |  |
|  |  |  |  |
| ADP 2^10^ -5M | L 0 | % | 50-150 |
| ADP 4^10^ -6M | L 0 | % | 32-100 |
| ADP 2^10^ -6M | L 0 | % | 16-70 |
| Collagen 200 micrgr/ml | L 0 | % | 50-150 |
| Arach Acid 500 micrgr/ml | L 0 | % | 50-150 |
| Platelet Rich Plasma | 285 | 10*3/mm*3 | 200-300 |
| BT | >10 | Min | 3-7 |
| Platelet count | 405 | 10*3/mm*3 | 150-450 |
|  |  |  |  |

Supplementary Table 1. Platelet aggregation and coagulation tests results

**Hematology (C.B.C)**

| Test | Result | Unit | Normal Range |
| --- | --- | --- | --- |
|  |  |  |  |
| WBC | 14.7 | 1000/ul | 35-11 |
| RBC | 4.27 | mil/ul | 4.2-5.4 |
| Hemoglobin | 10.4 | g/dL | 10-13.5 |
| Hematocrite | 34.7 | % | 37-53 |
| M.C.V | 81.3 | fL | 79-96 |
|  |  |  |  |
| M.C.H | 24.4 | pg | 27-33 |
| M.C.H.C | 30.0 | % | 33-36 |
| Platelets | 405 | 1000/ul | 150-450 |
| Platelet Morphology | - |  |  |
| RBC Morphology | Hypochromia (+) |  |  |
|  |  |  |  |

Supplementary Table 2. Hematology tests results

| Test | Result | Unit | Refrence value |
| --- | --- | --- | --- |
|  |  |  |  |
| W.B.C | 9.7 | %10^3/uL | 4.0-10.0 |
| R.B.C | 4.8 | %10^6/uL | 4.6-6.2 |
| HGB | 12.3 | g/dL |  |
| HCT | 36.4 | % | 38-47 |
| MCV | *76.3* | fl | 78-102 |
| MCH | 25.8 | pg | 27-35 |
| MCHC | 33.8 | g/dL | 32-37 |
|  |  |  |  |
|  |  |  |  |

Supplementary Table 3. Hematological tests before surgery

| Test | Result | Unit | Refrence value |
| --- | --- | --- | --- |
|  |  |  |  |
| PT | 12.3 | Sec | 11-14 |
| INR | 0.9 | - |  |
|  |  |  |  |

Supplementary Table 4. Coagulation tests
